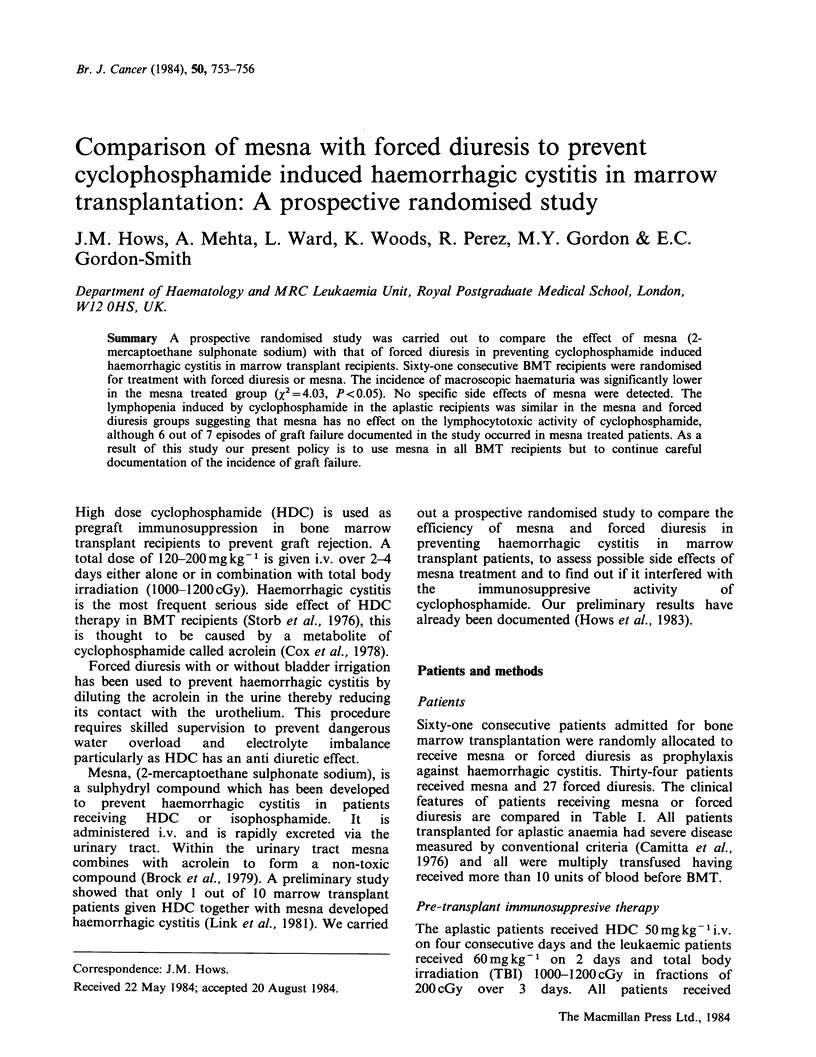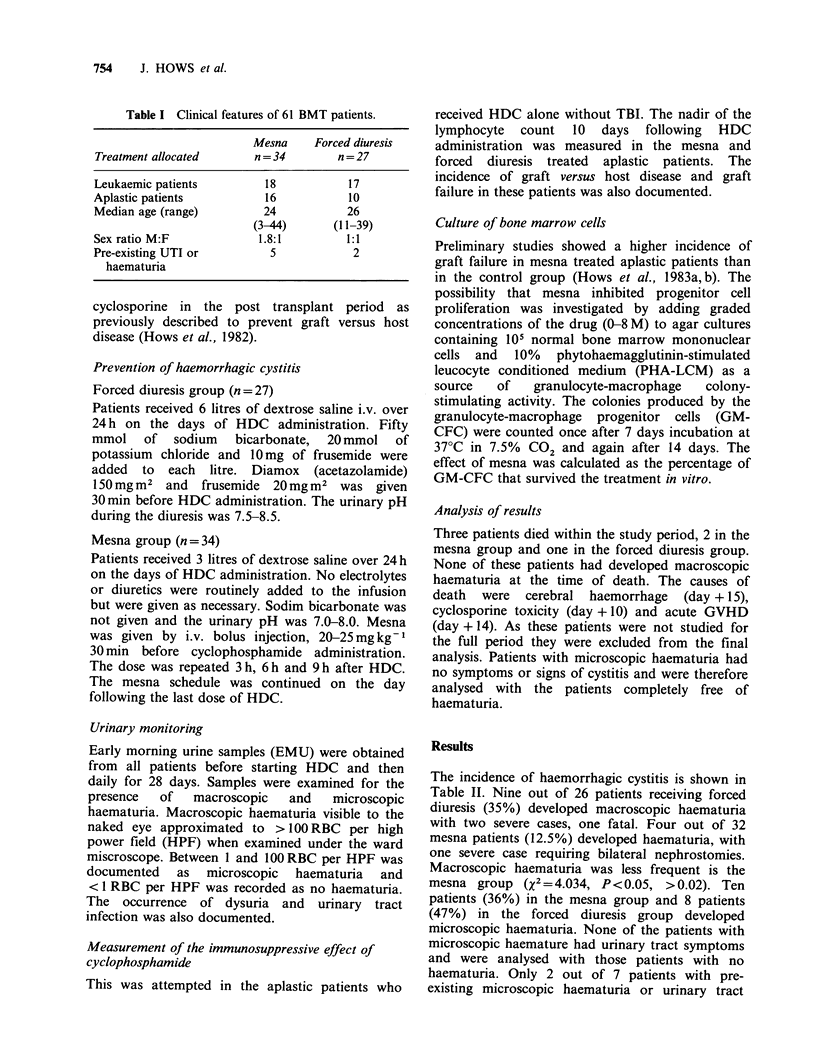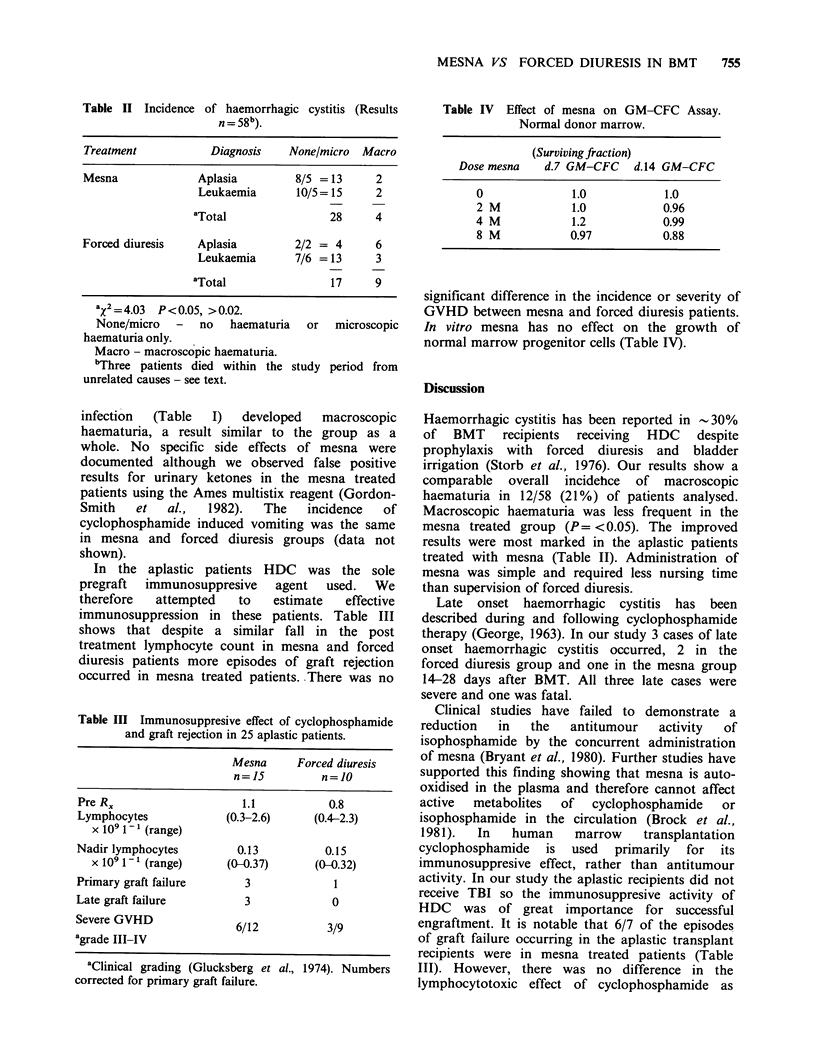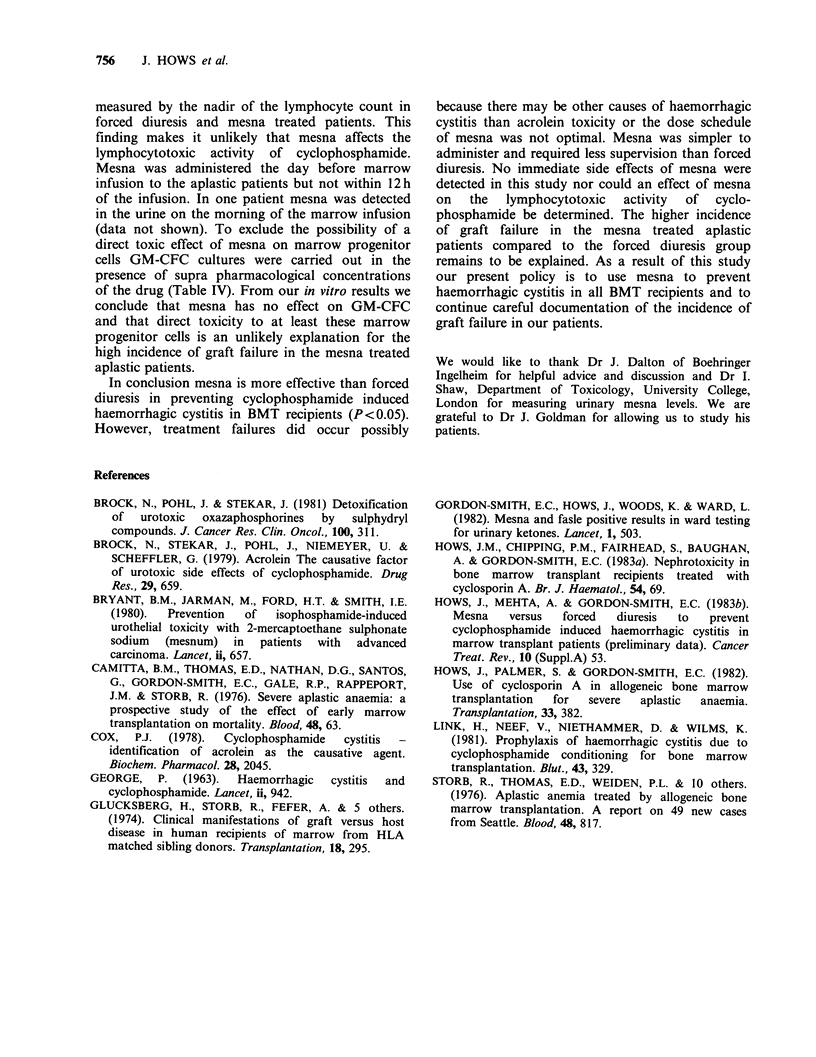# Comparison of mesna with forced diuresis to prevent cyclophosphamide induced haemorrhagic cystitis in marrow transplantation: a prospective randomised study.

**DOI:** 10.1038/bjc.1984.252

**Published:** 1984-12

**Authors:** J. M. Hows, A. Mehta, L. Ward, K. Woods, R. Perez, M. Y. Gordon, E. C. Gordon-Smith

## Abstract

A prospective randomised study was carried out to compare the effect of mesna (2-mercaptoethane sulphonate sodium) with that of forced diuresis in preventing cyclophosphamide induced haemorrhagic cystitis in marrow transplant recipients. Sixty-one consecutive BMT recipients were randomised for treatment with forced diuresis or mesna. The incidence of macroscopic haematuria was significantly lower in the mesna treated group (chi 2 = 4.03, P less than 0.05). No specific side effects of mesna were detected. The lymphopenia induced by cyclophosphamide in the aplastic recipients was similar in the mesna and forced diuresis groups suggesting that mesna has no effect on the lymphocytotoxic activity of cyclophosphamide, although 6 out of 7 episodes of graft failure documented in the study occurred in mesna treated patients. As a result of this study our present policy is to use mesna in all BMT recipients but to continue careful documentation of the incidence of graft failure.


Br. J. Cancer (1984), 50, 753–756

### Comparison of mesna with forced diuresis to prevent cyclophosphamide induced haemorrhagic cystitis in marrow transplantation: A prospective randomised study

J.M. Hows, A. Mehta, L. Ward, K. Woods, R. Perez, M.Y. Gordon & E.C. Gordon-Smith

*Department of Haematology and MRC Leukaemia Unit, Royal Postgraduate Medical School, London, W12 0HS, UK.*

**Summary** A prospective randomised study was carried out to compare the effect of mesna (2-mercaptoethane sulphonate sodium) with that of forced diuresis in preventing cyclophosphamide induced haemorrhagic cystitis in marrow transplant recipients. Sixty-one consecutive BMT recipients were randomised for treatment with forced diuresis or mesna. The incidence of macroscopic haematuria was significantly lower in the mesna treated group ($\chi^2=4.03$, $P<0.05$). No specific side effects of mesna were detected. The lymphopenia induced by cyclophosphamide in the aplastic recipients was similar in the mesna and forced diuresis groups suggesting that mesna has no effect on the lymphocytotoxic activity of cyclophosphamide, although 6 out of 7 episodes of graft failure documented in the study occurred in mesna treated patients. As a result of this study our present policy is to use mesna in all BMT recipients but to continue careful documentation of the incidence of graft failure.

High dose cyclophosphamide (HDC) is used as pregraft immunosuppression in bone marrow transplant recipients to prevent graft rejection. A total dose of 120–200 mg kg$^{-1}$ is given i.v. over 2–4 days either alone or in combination with total body irradiation (1000–1200 cGy). Haemorrhagic cystitis is the most frequent serious side effect of HDC therapy in BMT recipients (Storb *et al.*, 1976), this is thought to be caused by a metabolite of cyclophosphamide called acrolein (Cox *et al.*, 1978).

Forced diuresis with or without bladder irrigation has been used to prevent haemorrhagic cystitis by diluting the acrolein in the urine thereby reducing its contact with the urothelium. This procedure requires skilled supervision to prevent dangerous water overload and electrolyte imbalance particularly as HDC has an anti diuretic effect.

Mesna, (2-mercaptoethane sulphonate sodium), is a sulphydryl compound which has been developed to prevent haemorrhagic cystitis in patients receiving HDC or isophosphamide. It is administered i.v. and is rapidly excreted via the urinary tract. Within the urinary tract mesna combines with acrolein to form a non-toxic compound (Brock *et al.*, 1979). A preliminary study showed that only 1 out of 10 marrow transplant patients given HDC together with mesna developed haemorrhagic cystitis (Link *et al.*, 1981). We carried out a prospective randomised study to compare the efficiency of mesna and forced diuresis in preventing haemorrhagic cystitis in marrow transplant patients, to assess possible side effects of mesna treatment and to find out if it interfered with the immunosuppresive activity of cyclophosphamide. Our preliminary results have already been documented (Hows *et al.*, 1983).

Correspondence: J.M. Hows.

Received 22 May 1984; accepted 20 August 1984.

### Patients and methods

### *Patients*

Sixty-one consecutive patients admitted for bone marrow transplantation were randomly allocated to receive mesna or forced diuresis as prophylaxis against haemorrhagic cystitis. Thirty-four patients received mesna and 27 forced diuresis. The clinical features of patients receiving mesna or forced diuresis are compared in Table I. All patients transplanted for aplastic anaemia had severe disease measured by conventional criteria (Camitta *et al.*, 1976) and all were multiply transfused having received more than 10 units of blood before BMT.

### *Pre-transplant immunosuppresive therapy*

The aplastic patients received HDC 50 mg kg$^{-1}$ i.v. on four consecutive days and the leukaemic patients received 60 mg kg$^{-1}$ on 2 days and total body irradiation (TBI) 1000–1200 cGy in fractions of 200 cGy over 3 days. All patients received

The Macmillan Press Ltd., 1984

**Table I** Clinical features of 61 BMT patients.

| *Treatment allocated* | *Mesna* $n=34$ | *Forced diuresis* $n=27$ |
|---|---|---|
| Leukaemic patients | 18 | 17 |
| Aplastic patients | 16 | 10 |
| Median age (range) | 24 (3–44) | 26 (11–39) |
| Sex ratio M:F | 1.8:1 | 1:1 |
| Pre-existing UTI or haematuria | 5 | 2 |

cyclosporine in the post transplant period as previously described to prevent graft versus host disease (Hows *et al.*, 1982).

*Prevention of haemorrhagic cystitis*

Forced diuresis group ($n=27$)

Patients received 6 litres of dextrose saline i.v. over 24 h on the days of HDC administration. Fifty mmol of sodium bicarbonate, 20 mmol of potassium chloride and 10 mg of frusemide were added to each litre. Diamox (acetazolamide) 150 mg m$^2$ and frusemide 20 mg m$^2$ was given 30 min before HDC administration. The urinary pH during the diuresis was 7.5–8.5.

Mesna group ($n=34$)

Patients received 3 litres of dextrose saline over 24 h on the days of HDC administration. No electrolytes or diuretics were routinely added to the infusion but were given as necessary. Sodim bicarbonate was not given and the urinary pH was 7.0–8.0. Mesna was given by i.v. bolus injection, 20–25 mg kg$^{-1}$ 30 min before cyclophosphamide administration. The dose was repeated 3 h, 6 h and 9 h after HDC. The mesna schedule was continued on the day following the last dose of HDC.

*Urinary monitoring*

Early morning urine samples (EMU) were obtained from all patients before starting HDC and then daily for 28 days. Samples were examined for the presence of macroscopic and microscopic haematuria. Macroscopic haematuria visible to the naked eye approximated to >100 RBC per high power field (HPF) when examined under the ward miscroscope. Between 1 and 100 RBC per HPF was documented as microscopic haematuria and <1 RBC per HPF was recorded as no haematuria. The occurrence of dysuria and urinary tract infection was also documented.

*Measurement of the immunosuppressive effect of cyclophosphamide*

This was attempted in the aplastic patients who received HDC alone without TBI. The nadir of the lymphocyte count 10 days following HDC administration was measured in the mesna and forced diuresis treated aplastic patients. The incidence of graft *versus* host disease and graft failure in these patients was also documented.

*Culture of bone marrow cells*

Preliminary studies showed a higher incidence of graft failure in mesna treated aplastic patients than in the control group (Hows *et al.*, 1983a, b). The possibility that mesna inhibited progenitor cell proliferation was investigated by adding graded concentrations of the drug (0–8 M) to agar cultures containing $10^5$ normal bone marrow mononuclear cells and 10% phytohaemagglutinin-stimulated leucocyte conditioned medium (PHA-LCM) as a source of granulocyte-macrophage colony-stimulating activity. The colonies produced by the granulocyte-macrophage progenitor cells (GM-CFC) were counted once after 7 days incubation at 37°C in 7.5% $CO_2$ and again after 14 days. The effect of mesna was calculated as the percentage of GM-CFC that survived the treatment *in vitro*.

*Analysis of results*

Three patients died within the study period, 2 in the mesna group and one in the forced diuresis group. None of these patients had developed macroscopic haematuria at the time of death. The causes of death were cerebral haemorrhage (day + 15), cyclosporine toxicity (day + 10) and acute GVHD (day + 14). As these patients were not studied for the full period they were excluded from the final analysis. Patients with microscopic haematuria had no symptoms or signs of cystitis and were therefore analysed with the patients completely free of haematuria.

### Results

The incidence of haemorrhagic cystitis is shown in Table II. Nine out of 26 patients receiving forced diuresis (35%) developed macroscopic haematuria with two severe cases, one fatal. Four out of 32 mesna patients (12.5%) developed haematuria, with one severe case requiring bilateral nephrostomies. Macroscopic haematuria was less frequent is the mesna group ($\chi^2=4.034$, $P<0.05$, $>0.02$). Ten patients (36%) in the mesna group and 8 patients (47%) in the forced diuresis group developed microscopic haematuria. None of the patients with microscopic haemature had urinary tract symptoms and were analysed with those patients with no haematuria. Only 2 out of 7 patients with pre-existing microscopic haematuria or urinary tract

**Table II** Incidence of haemorrhagic cystitis (Results $n = 58^b$).

| *Treatment* | *Diagnosis* | *None/micro* | *Macro* |
|---|---|---|---|
| Mesna | Aplasia | 8/5 = 13 | 2 |
| | Leukaemia | 10/5 = 15 | 2 |
| | [a]Total | 28 | 4 |
| Forced diuresis | Aplasia | 2/2 = 4 | 6 |
| | Leukaemia | 7/6 = 13 | 3 |
| | [a]Total | 17 | 9 |

[a]$\chi^2 = 4.03$ $P < 0.05$, $> 0.02$.
None/micro – no haematuria or microscopic haematuria only.
Macro – macroscopic haematuria.
[b]Three patients died within the study period from unrelated causes – see text.

infection (Table I) developed macroscopic haematuria, a result similar to the group as a whole. No specific side effects of mesna were documented although we observed false positive results for urinary ketones in the mesna treated patients using the Ames multistix reagent (Gordon-Smith *et al.*, 1982). The incidence of cyclophosphamide induced vomiting was the same in mesna and forced diuresis groups (data not shown).

In the aplastic patients HDC was the sole pregraft immunosuppresive agent used. We therefore attempted to estimate effective immunosuppression in these patients. Table III shows that despite a similar fall in the post treatment lymphocyte count in mesna and forced diuresis patients more episodes of graft rejection occurred in mesna treated patients. There was no

**Table III** Immunosuppresive effect of cyclophosphamide and graft rejection in 25 aplastic patients.

| | *Mesna* $n = 15$ | *Forced diuresis* $n = 10$ |
|---|---|---|
| Pre $R_x$ Lymphocytes $\times 10^9 l^{-1}$ (range) | 1.1 (0.3–2.6) | 0.8 (0.4–2.3) |
| Nadir lymphocytes $\times 10^9 l^{-1}$ (range) | 0.13 (0–0.37) | 0.15 (0–0.32) |
| Primary graft failure | 3 | 1 |
| Late graft failure | 3 | 0 |
| Severe GVHD [a]grade III–IV | 6/12 | 3/9 |

[a]Clinical grading (Glucksberg *et al.*, 1974). Numbers corrected for primary graft failure.

**Table IV** Effect of mesna on GM–CFC Assay. Normal donor marrow.

| *Dose mesna* | (*Surviving fraction*) *d.7 GM–CFC* | *d.14 GM–CFC* |
|---|---|---|
| 0 | 1.0 | 1.0 |
| 2 M | 1.0 | 0.96 |
| 4 M | 1.2 | 0.99 |
| 8 M | 0.97 | 0.88 |

significant difference in the incidence or severity of GVHD between mesna and forced diuresis patients. *In vitro* mesna has no effect on the growth of normal marrow progenitor cells (Table IV).

### Discussion

Haemorrhagic cystitis has been reported in ~30% of BMT recipients receiving HDC despite prophylaxis with forced diuresis and bladder irrigation (Storb *et al.*, 1976). Our results show a comparable overall incidehce of macroscopic haematuria in 12/58 (21%) of patients analysed. Macroscopic haematuria was less frequent in the mesna treated group ($P = < 0.05$). The improved results were most marked in the aplastic patients treated with mesna (Table II). Administration of mesna was simple and required less nursing time than supervision of forced diuresis.

Late onset haemorrhagic cystitis has been described during and following cyclophosphamide therapy (George, 1963). In our study 3 cases of late onset haemorrhagic cystitis occurred, 2 in the forced diuresis group and one in the mesna group 14–28 days after BMT. All three late cases were severe and one was fatal.

Clinical studies have failed to demonstrate a reduction in the antitumour activity of isophosphamide by the concurrent administration of mesna (Bryant *et al.*, 1980). Further studies have supported this finding showing that mesna is auto-oxidised in the plasma and therefore cannot affect active metabolites of cyclophosphamide or isophosphamide in the circulation (Brock *et al.*, 1981). In human marrow transplantation cyclophosphamide is used primarily for its immunosuppresive effect, rather than antitumour activity. In our study the aplastic recipients did not receive TBI so the immunosuppresive activity of HDC was of great importance for successful engraftment. It is notable that 6/7 of the episodes of graft failure occurring in the aplastic transplant recipients were in mesna treated patients (Table III). However, there was no difference in the lymphocytotoxic effect of cyclophosphamide as measured by the nadir of the lymphocyte count in forced diuresis and mesna treated patients. This finding makes it unlikely that mesna affects the lymphocytotoxic activity of cyclophosphamide. Mesna was administered the day before marrow infusion to the aplastic patients but not within 12 h of the infusion. In one patient mesna was detected in the urine on the morning of the marrow infusion (data not shown). To exclude the possibility of a direct toxic effect of mesna on marrow progenitor cells GM-CFC cultures were carried out in the presence of supra pharmacological concentrations of the drug (Table IV). From our *in vitro* results we conclude that mesna has no effect on GM-CFC and that direct toxicity to at least these marrow progenitor cells is an unlikely explanation for the high incidence of graft failure in the mesna treated aplastic patients.

In conclusion mesna is more effective than forced diuresis in preventing cyclophosphamide induced haemorrhagic cystitis in BMT recipients ($P<0.05$). However, treatment failures did occur possibly because there may be other causes of haemorrhagic cystitis than acrolein toxicity or the dose schedule of mesna was not optimal. Mesna was simpler to administer and required less supervision than forced diuresis. No immediate side effects of mesna were detected in this study nor could an effect of mesna on the lymphocytotoxic activity of cyclophosphamide be determined. The higher incidence of graft failure in the mesna treated aplastic patients compared to the forced diuresis group remains to be explained. As a result of this study our present policy is to use mesna to prevent haemorrhagic cystitis in all BMT recipients and to continue careful documentation of the incidence of graft failure in our patients.

We would like to thank Dr J. Dalton of Boehringer Ingelheim for helpful advice and discussion and Dr I. Shaw, Department of Toxicology, University College, London for measuring urinary mesna levels. We are grateful to Dr J. Goldman for allowing us to study his patients.

**References**

BROCK, N., POHL, J. & STEKAR, J. (1981) Detoxification of urotoxic oxazaphosphorines by sulphydryl compounds. *J. Cancer Res. Clin. Oncol.*, **100**, 311.

BROCK, N., STEKAR, J., POHL, J., NIEMEYER, U. & SCHEFFLER, G. (1979). Acrolein The causative factor of urotoxic side effects of cyclophosphamide. *Drug Res.*, **29**, 659.

BRYANT, B.M., JARMAN, M., FORD, H.T. & SMITH, I.E. (1980). Prevention of isophosphamide-induced urothelial toxicity with 2-mercaptoethane sulphonate sodium (mesnum) in patients with advanced carcinoma. *Lancet*, **ii**, 657.

CAMITTA, B.M., THOMAS, E.D., NATHAN, D.G., SANTOS, G., GORDON-SMITH, E.C., GALE, R.P., RAPPEPORT, J.M. & STORB, R. (1976). Severe aplastic anaemia: a prospective study of the effect of early marrow transplantation on mortality. *Blood*, **48**, 63.

COX, P.J. (1978). Cyclophosphamide cystitis – identification of acrolein as the causative agent. *Biochem. Pharmacol.* **28**, 2045.

GEORGE, P. (1963). Haemorrhagic cystitis and cyclophosphamide. *Lancet*, **ii**, 942.

GLUCKSBERG, H., STORB, R., FEFER, A. & 5 others. (1974). Clinical manifestations of graft versus host disease in human recipients of marrow from HLA matched sibling donors. *Transplantation*, **18**, 295.

GORDON-SMITH, E.C., HOWS, J., WOODS, K. & WARD, L. (1982). Mesna and fasle positive results in ward testing for urinary ketones. *Lancet*, **1**, 503.

HOWS, J.M., CHIPPING, P.M., FAIRHEAD, S., BAUGHAN, A. & GORDON-SMITH, E.C. (1983*a*). Nephrotoxicity in bone marrow transplant recipients treated with cyclosporin A. *Br. J. Haematol.*, **54**, 69.

HOWS, J., MEHTA, A. & GORDON-SMITH, E.C. (1983*b*). Mesna versus forced diuresis to prevent cyclophosphamide induced haemorrhagic cystitis in marrow transplant patients (preliminary data). *Cancer Treat. Rev.*, **10** (Suppl.A) 53.

HOWS, J., PALMER, S. & GORDON-SMITH, E.C. (1982). Use of cyclosporin A in allogeneic bone marrow transplantation for severe aplastic anaemia. *Transplantation*, **33**, 382.

LINK, H., NEEF, V., NIETHAMMER, D. & WILMS, K. (1981). Prophylaxis of haemorrhagic cystitis due to cyclophosphamide conditioning for bone marrow transplantation. *Blut.*, **43**, 329.

STORB, R., THOMAS, E.D., WEIDEN, P.L. & 10 others. (1976). Aplastic anemia treated by allogeneic bone marrow transplantation. A report on 49 new cases from Seattle. *Blood*, **48**, 817.